# Identification, Cloning, and Expression of L-Amino Acid Oxidase from Marine *Pseudoalteromonas* sp. B3

**DOI:** 10.1155/2014/979858

**Published:** 2014-01-09

**Authors:** Zhiliang Yu, Ning Zhou, Hua Qiao, Juanping Qiu

**Affiliations:** College of Biological and Environmental Engineering, Zhejiang University of Technology, Hangzhou 310014, China

## Abstract

L-amino acid oxidase (LAAO) is attracting more attentions due to its broad and important biological functions. Recently, an LAAO-producing marine microorganism (strain B3) was isolated from the intertidal zone of Dinghai sea area, China. Physiological, biochemical, and molecular identifications together with phylogenetic analysis congruously suggested that it belonged to the genus *Pseudoalteromonas*. Therefore, it was designated as *Pseudoalteromonas* sp. B3. Its capability of LAAO production was crossly confirmed by measuring the products of H_2_O_2_, a-keto acids, and NH_4_^+^ in oxidization reaction. Two rounds of PCR were performed to gain the entire B3-LAAO gene sequence of 1608 bps in length encoding for 535 amino acid residues. This deduced amino acid sequence showed 60 kDa of the calculated molecular mass, supporting the SDS-PAGE result. Like most of flavoproteins, B3-LAAO also contained two conserved typical motifs, GG-motif and **β** **α** **β**-dinucleotide-binding domain motif. On the other hand, its unique substrate spectra and sequence information suggested that B3-LAAO was a novel LAAO. Our results revealed that it could be functionally expressed in *E. coli* BL21(DE3) using vectors, pET28b(+) and pET20b(+). However, compared with the native LAAO, the expression level of the recombinant one was relatively low, most probably due to the formation of inclusion bodies. Several solutions are currently being conducted in our lab to increase its expression level.

## 1. Introduction

L-amino acid oxidase (LAAO; EC 1.4.3.2) is usually dimeric flavoprotein containing a noncovalently bound FAD molecule as cofactor for each subunit. It is able to catalyze the stereospecific oxidative deamination of L-amino acids to the corresponding a-keto acids with release of NH_4_^+^ and H_2_O_2_ which is believed to be associated with its biological activities including inducing apoptosis, cytotoxicity, edema, hemolysis, hemorrhage, inducing or inhibiting platelet aggregation, and parasite-killing and antimicrobial activities [1]. When H_2_O_2_ is not degraded by catalase, it can cause a decarboxylation of the a-keto acid to the corresponding carboxylic acid.

This enzyme is widely distributed in different sources including snake venom [2], sea hare [3], insect drugs [4], algae [5], and microorganisms [6, 7]. So far, the LAAO from snake venom is the best characterized member of this enzyme family with respect to not only its toxicology but also its biochemistry, physiology, and medicine. In contrast, very little is known about LAAO from marine microorganism. Although various LAAO-coding sequences have been published to reveal that LAAO family members commonly have flavin as coenzyme and possess two conserved and characteristic sequence motifs, “GG” motif (RxGGRxxS/T) and dinucleotide-binding (DMB) motif (*β*-strand/*α*-helix/*β*-strand) [8], only some heterologous expression systems have been reported hitherto, probably due to the requirement of posttranslational modification of LAAO. In 2003, the first bacterial heterologous expression system for an LAAO from *Rhodococcus opacus *was reported. The *lao*-gene from* R. opacus *was cloned into *Escherichia coli* and *Streptomyces lividans *expression vectors. It was found that the expression in* E. coli* resulted in the accumulation of insoluble protein, but* S. lividans* was a suitable host for the heterologous expression of LAAO [9]. However, in 2008, the *lao*-gene from *Streptococcus oligofermentans *was successfully cloned and overexpressed in *E. coli* [10]. The LAAO isolated from sea hare was also functionally expressed in *E. coli*, but the expression level for soluble recombinant LAAO was relatively low, only ca. 0.2 mg/L culture medium [11]. To our knowledge, the expression of ophidian LAAO has rarely been achieved, except the successful expression of active recombinant LAAO in the methylotrophic yeast* Pichia pastoris* [12]. To date, the heterologous expression of LAAO is still a big challenge.

In this study, a yellow-pigmented LAAO-producing marine bacterial strain designated as *Pseudoalteromonas* sp. B3 on the basis of the physiological, biochemical, and molecular analysis was isolated. After retrieval of coding sequence, LAAO from *Pseudoalteromonas *sp. B3 (B3-LAAO) was cloned and functionally expressed in *E. coli* strain BL21 (DE3). This communication laid the foundation for further studies on enzymatic properties, structure, biological function, and application of B3-LAAO.

## 2. Materials and Methods

### 2.1. Sample Collection and Colony Isolation

The intertidal sediment samples were obtained from Dinghai sea area, Zhoushan, China (30.03°N, 122.11°E). Each sample was collected at 50 to 100 cm depth below the sea surface. The samples were placed in special presterilized plastic bottles and brought to the lab in aseptic condition. After serial dilution (up to 10^−6^ dilution) using sterilized sea water, 100 *μ*L of each diluted sample was plated on PDA (potato 200 g/L, sucrose 10 g/L, sea salt 30 g/L, 20 g/L agar), Gause's NO. 1 (soluble starch 20 g/L, NaCl 0.5 g/L, K_2_HPO_4_ 0.5 g/L, FeSO_4_ 0.01 g/L, MgSO_4_·7H_2_O 0.5 g/L, KNO_3_ 1 g/L, sea salt 30 g/L, 20 g/L agar, pH 7.2–7.4) and MM medium (yeast extract 3 g/L, peptone 5 g/L, sea salt 30 g/L, 20 g/L agar, pH 7.2–7.4) and separately incubated at either 28°C or 25°C for 2–7 days as necessary. To make broth medium, agar was absent. The isolated colonies were purified by streak-plate technique. Then, each pure isolate was cultured in 50 mL of broth medium and culture supernatant was collected after centrifuge at 8000 rpm for 10 min at 4°C for subsequent LAAO activity screening.

### 2.2. Screening of LAAO-Producing Microorganism

LAAO activity produced by isolate was detected by measuring its oxidization productions of L-amino acids including H_2_O_2_, a-keto acid, and NH_4_^+^. (1) H_2_O_2_ measurement: 1.5 mL of resultant culture supernatant was mixed with 1.5 mL of 10 mM L-Leu for oxidization reaction at 37°C for 30 min. Then, the produced H_2_O_2_ was measured using Amplex Red Hydrogen Peroxide/Peroxidase Assay kit (Invitrogen, USA) according to manufacture's instruction. (2) a-Keto acid measurement: 1.5 mL of culture supernatant was mixed with 1.5 mL of 10 mM L-Leu for oxidization reaction at 37°C for 30 min. Then, as reported [13], the produced a-keto leucine was detected using 2,4-dinitrophenylhydrazine (DNP) which can react with carbonyl group to generate brown-red dinitrophenylhydrazone. Briefly, 550 *μ*L of detection solution was mixed with 450 *μ*L of 20% trichloroacetic acid and kept at room temperature for 30 min. Next, 200 *μ*L of 20 mM DNP was added and the mixture was incubated at room temperature for 15 min. The reaction was terminated by adding 4 mL of 0.8 M NaOH. Finally, the mixture was centrifuged at 12,000 rpm for 10 min and the absorbance of the supernatant was measured at 520 nm. (3) NH_4_^+^ detection: 25 mL of resultant culture supernatant was mixed with 25 mL of 10 mM L-Leu in 250 mL glass flask for oxidization reaction at 37°C for 30 min. Then, 1 mL of 1 M NaOH was added and paper pH indicator was put above the glass flask. After shaking at 120 rpm for 60 min at room temperature, the color change of paper pH indicator due to the NH_3_ release was observed.

### 2.3. Molecular Taxonomy and Phylogenetic Analysis

The genomic DNA of LAAO-producing microorganism was extracted by bacterial DNA isolation method [14]. 16S rDNA gene was amplified in 50 *μ*L containing 37 *μ*L of ddH_2_O, 5 *μ*L of 10×Easy *Taq* buffer, 4 *μ*L of 2.5 mM dNTPs, 100 nM forward primer 27F (5′-GAGTTTGATCCTGGCTCAG-3′), 100 nM reverse primer 1527R (5′-AGAAAGGAGGTGATCCAGCC-3′), 1 ng genomic DNA, and 1 U *Taq* DNA polymerase with denaturation at 94°C for 5 min followed by 30 cycles of 1 min at 94°C, 50 s at 55°C, 90 s at 72°C, and a final 10 min extension at 72°C. At the end of reaction, PCR product was cooled to 4°C to await further use. After size confirmation on 1.0% agarose gel, the PCR product was sent to Sangon Biotech (Shanghai) Co. Ltd for sequencing of 16S rDNA. The similarity and homology of 16S rDNA gene sequence was analyzed using BLAST search available in genbank of NCBI. The DNA sequences were aligned and phylogenetic tree was constructed by neighbor joining method with bootstrap trials 1000 using MegaV4.0.2 software.

### 2.4. Physiological and Biochemical Characterization

The ability of the isolate to utilize various carbon and nitrogen sources and other physiological and biochemical properties was studied according to the recommendation in “The Manual of Systematic Methods of Determinative Bacterial.”

### 2.5. Retrieval of LAAO Gene

To retrieve the full length of *lao*-gene sequence from isolated microbial producer, two rounds of PCR were performed. For the first round PCR, the 6 degenerate primers were designed based on the conserved regions after alignment of LAAO sequences from general LAAO-producing marine microorganisms (see Table 1) including *Comamonas testosteroni *KF-1 (ZP_03541004), *Dinoroseobacter shibae* DFL 12 (ABV95616), and *Caulobacter* sp. K31 (YP_001683007) and LAAO-producing *Pseudoalteromonas* microorganisms (see Table 2) including *Pseudoalteromonas haloplanktis* TAC125 (YP_339251), *Pseudoalteromonas tunicata *D2 (ZP_01132853), *Pseudoalteromonas atlantica *T6c (YP_662716), and *Pseudoalteromonas *sp. SM9913 (YP_004069407). ZZ-1, ZZ-2 and ZZ-3 were forward primers and ZF-1, ZF-2, and ZF-3 are reverse ones. As required, these 6 degenerate primers combined with each other to form 9 sets of forward and reverse primer pairs. PCR amplification was performed with genomic DNA of LAAO-producer as template and PrimeSTAR HS DNA polymerase (TaKaRa, Dalian, China) as polymerase. The Touch-down PCR consisted of denaturation at 94°C for 5 min, 15 cycle of 30 s at 94°C, 30 s at 64°C (−1°C/cycle), and 1 min at 72°C, followed by another 20 cycles of 30 s at 94°C, 30 s at 48°C, and 1 min at 72°C and a final 10 min extension at 72°C. After size confirmation on 1% agarose gel and gel extraction (Qiagen, CA, USA), the PCR product with expected size was cloned into pMD19-T simple vector (TaKaRa, Dalian, China) and sequenced by Sangon Biotech (Shanghai) Co. Ltd.

To obtain the entire *lao*-gene, the second round of inverse PCR (using nested-PCR technique) was employed to amplify the flanked gene sequences (5′ and 3′ regions). As shown in Table 3, two nested specific primer sets, 5F-1 (1st), 5F-2 (2nd), and 5F-3 (3rd) for 5′ flanked region and 3Z-1 (1st), 3Z-2 (2nd), and 3Z-3 (3rd) for 3′ flanked region, were designed based on the intermediated *lao*-gene sequence obtained in the first round of PCR. Nine arbitrary primers (S-1, S-2, S-3, S-4, S-5, S-6, B0043, B0043-9, and B0043-10) (see Table 3) were purchased from Invitrogen (USA) and each arbitrary primer was used to pair with nested specific primer to form forward and reverse primer set. For either 5′ or 3′ flanked region of *lao*-gene, a total of 27 nested-PCR amplification reactions were performed with denaturation at 94°C for 5 min, 6 cycles of 30 s at 94°C, 50 s at 35°C, and 2 min at 72°C, followed by another 30 cycles of 60 s at 94°C, 50 s at 55°C, and 2 min at 72°C and a final 10 min extension at 72°C. The desired PCR product was purified using a gel extraction kit (Qiagen, CA, USA) and cloned into pMD19-T simple vector (TaKaRa, Dalian, China). After sequencing by Sangon Biotech (Shanghai) Co. Ltd, the contigs of 5′ flanked fragment, intermediated fragment, and 3′ flanked fragment were assembled using “Cap Conting Assembly” of BioEdit software V5.2.

### 2.6. Cloning and Expression of LAAO in *E. coli* BL21(DE3)

The complete *lao*-gene was amplified using a pair of forward primer (B3laaoF, 5′-CGCGGATCCTATGAAAGAACAAGTTC-3′, with a *Bam*H I restriction site as underlined) and reverse primer (B3laaoR, 5′-CCCAAGCTTACGTTTGATTTTACTGG-3′, with a *Hin*d III restriction site). *Pfu* DNA polymerase (Promega) was used in a PCR to amplify the 1,608 bps *lao*-gene. The desired PCR product was subsequently cloned into pMD19-T simple vector, and the *lao*-gene sequence was confirmed by *Bam*H I and *Hin*d III digestion and direct DNA sequencing. After *Bam*H I-*Hin*d III digestion, the released *lao*-gene was subsequently cloned into the *Bam*H I-*Hin*d III restriction sites of expression vectors, pET-28b(+) and pET-20b(+) (Novagen, USA), to generate pET-28b(+)-*lao* and pET-20b(+)-*lao*, respectively. The inserted gene was confirmed by DNA sequencing (Sangon Biotech (Shanghai) Co. Ltd.). The plasmids, pET-28b(+)-*lao* and pET-20b(+)-*lao*, inserted with a PCR-amplified *lao-*gene was separately transformed into *E. coli *BL21(DE3) (Novagen) cells and cultured in LB medium supplemented with 50 *μ*g/mL kanamycin and 100 *μ*g/mL ampicillin. Cells were grown at 37°C to an OD_600_ of 0.4 to 0.6. Overproduction of the LAAO protein was induced by addition of 1 mM isopropyl-*β*-D-thiogalactopyranoside (IPTG) and 0.1 mM FAD together with 0.1 mM Zn^2+^ at 25°C for 6 h.

After expression, the cells from 1.5 mL of culture were harvested by a centrifugation at 6,000 rpm for 5 min at 4°C. Then, the cell pellets were resuspended in 100 *μ*L of ddH_2_O and mixed with 25 *μ*L of 4-fold sample loading buffer (1.0 M Tris-HCl, pH 6.8, 10% SDS, 20% *β*-mercaptoethanol, 50% glycerol, 1% bromophenol blue). After boiling for 5 min, 30 *μ*L of resultant sample mixture was subjected to SDS-polyacrylamide gel (SDS-PAGE) with a stacking gel of 5% and separation gel of 12%, as described [15], and the number and molecular weight of the expressed LAAO were determined.

### 2.7. Assay of the Expressed LAAO Activity

The expressed LAAO activity was determined by measuring the produced H_2_O_2_ with Prussian blue agar assay [16]. In brief, 25 mL of the induced cell culture was mixed with 25 mL of 10 mM L-Leu for oxidation reaction at 37°C with shaking at 120 rpm. After incubation for 1 h, 50 *μ*L of resultant oxidation mixture was subjected to the circular well with diameter of 6 mm on Prussian blue agar (1.0 g/L FeCl_3_·6H_2_O, 1.0 g/L potassium hexacyanoferrate (III), 2% agar, pH 7.5). After incubation for 30 min at room temperature, the color development resulted from Prussian blue formation due to the H_2_O_2_ production was monitored.

## 3. Results and Discussion

### 3.1. Isolation of LAAO-Producing Strain

Based on morphological identification, a total of 157 pure isolates, 32 from PDA medium, 51 from Gause's NO. 1 medium, and 74 from MM medium, were obtained. Out of those 157 isolates subjected to LAAO-producing screening, only one isolate (strain B3) gave the capability to produce LAAO, as determined and verified below.

**Figure 1:**
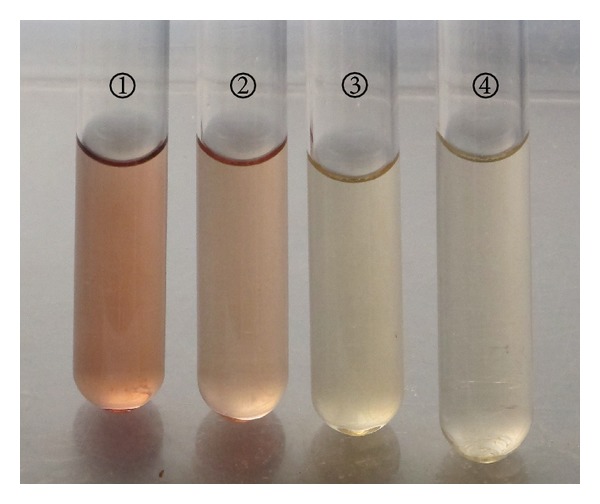
Detection of a-keto acid production using 2,4-dinitrophenylhydrazine (DNP) [13]. *①* positive control: *α*-keto leucine in MM medium (OD520 = 0.232); *②* culture supernatant of strain B3 with substrate L-Leu (OD520 = 0.198); *③* culture supernatant of strain B3 without substrate L-Leu (OD520 = 0.032); *④* boiling-denatured culture supernatant of strain B3 with substrate L-Leu (OD520 = 0.045).

**Figure 2:**
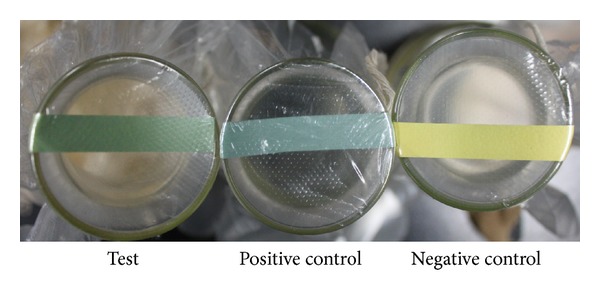
Determination of the presence of NH_4_^+^ after addition of NaOH using paper pH indicator. Test: culture supernatant of strain B3 with substrate L-Leu; positive control: ammonia solution; negative control: culture supernatant of strain B3 without substrate L-Leu.

First, LAAOs are able to catalyze L-amino acids to release H_2_O_2_ which can be detected using Amplex Red Hydrogen Peroxide/Peroxidase Assay kit. As shown in Table 4, almost no H_2_O_2_ was detected from either the culture supernatant of strain B3 without L-Leu (Test 2) or the denatured culture supernatant by boiling for 10 minutes followed by addition of L-Leu (Test 3). In contrast, decent amount of H_2_O_2_ (1.84 mM) was measured from the culture supernatant of strain B3 with L-Leu (Test 1), meaning that the culture supernatant of strain B3 can use L-Leu to release H_2_O_2_. All these findings indicate that strain B3 may bear the ability to produce LAAO. Second, as carbonyl derivative, a-keto acid can react with 2,4-dinitrophenylhydrazine (DNP) to generate brown-red dinitrophenylhydrazone with maximum absorbance at 520 nm [13]. As shown in Figure 1, the culture supernatant of strain B3 without L-Leu gave flaxen and OD_520_ was low, displaying the similar result as the denatured culture supernatant of strain B3 with L-Leu. In contrast, both the a-keto leucine (positive control) and the oxidation solution of L-Leu by the culture supernatant of strain B3 had brown-red color with high OD_520_ value. All these findings indicate that L-Leu can be oxidized by culture supernatant of strain B3 to generate the corresponding a-keto acid. Therefore, strain B3 can produce LAAO. Third, in the presence of strong base like NaOH, NH_4_^+^ in solution will become unstable to release NH_3_ which can easily be detected by paper pH indicator. The results in Figure 2 showed that after addition of NaOH, the culture supernatant of strain B3 without L-Leu (negative control) did not cause the color change of the paper pH indicator, maintaining the original yellow. In contrast, like the positive control (ammonia solution), the oxidization solution of L-Leu by the culture supernatant of strain B3 caused the color change of paper pH indicator from original yellow to absinthe-green. All these findings indicate that L-Leu can be used by the culture supernatant of strain B3 to release NH_4_^+^, supporting that strain B3 can produce LAAO.

Our quantitative and qualitative measurements together clearly showed that the culture supernatant of strain B3 can specifically oxidize L-Leu to yield H_2_O_2_, a-keto leucine, and NH_4_^+^, thus crossly supporting that the isolate B3 can produce LAAO. Among these three detection methods, H_2_O_2_ assay kit and DNP assay are quantitative methods with very high sensitivity, whereas paper pH indicator can only be used for qualitative measurement with low sensitivity. On the other hand, compared with the former two methods, the latter one gives advantages including convenience, no requirement of instrument, and cost-effectiveness, thus ideal for the first-step screening of LAAO-producing microorganisms.

### 3.2. Brief Characterization of Strain B3

**Figure 3:**
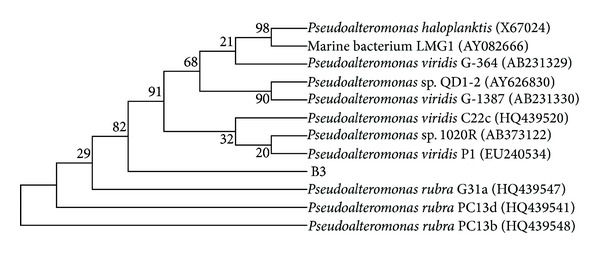
Phylogenetic tree of strain B3 based on 16S rDNA. The numbers at the nodes indicate the bootstrap level based on the analysis of 1000 resampled data sets.

A pair of primers, 27F and 1527R, were successfully used to amplify about 1.5 kb 16S rDNA gene from strain B3. After sequencing, a total of 1396 bp were gained. The Blast analysis of 16S rDNA at the NCBI website together with the constructed phylogenetic tree (see Figure 3) indicated that strain B3 was very proximal to *Pseudoalteromonas* spp. with the highest identity of 98.5% to *Pseudoalteromonas viridis*. On MM medium plate, strain B3 exhibited yellow smooth round. The Gram-staining reaction showed that it was Gram-negative (photo not shown). The physiological and biochemical analysis revealed that it was positive to starch hydrolysis, indole test, citrate utilization, and oxidase test and negative to lysine decarboxylation enzyme, half a solid agar, gelatin hydrolysis, H_2_S production, acetamide, V-P test, methyl red test, and melezitose monohydrate. In addition, it can consume galactose, glucose, lactose, fructose, maltose, and rhamnose as carbon source and (NH_4_)_2_HPO_4_, KNO_3_, arginine, methionine, glycine, and tyrosine as nitrogen sources for growth. Therefore, it was designated as *Pseudoalteromonas* sp. B3.

### 3.3. Retrieval of *lao* Gene and Characterization of the Deduced Amino Acid Sequence

**Figure 4:**
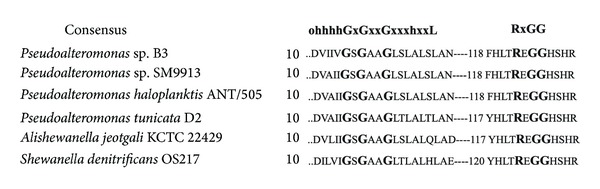
Sequence alignment between B3-LAAO and several homologous LAAOs. The highly conserved motifs, dinucleotide-binding domain motif ohhhhGxGxxGxxxhxxL for FAD binding where o stands for a polar or charged residue and h for a hydrophobic residue, and characteristic motif RxGG, are indicated.

Among 9 sets of primer pairs, only one pair from ZZ-2 and ZF-2 gave the desired PCR-amplified product with around 650 bps in size in the first round of PCR. After sequencing, 654 bps of intermediated LAAO-coding sequence from *Pseudoalteromonas *sp. B3 was obtained to show 80% identity to L-aspartate oxidase-coding gene from *Pseudoalteromonas *sp. SM9913. In the second round of inverse PCR, only arbitrary primer S-2 was combined with one set of nested specific primers (5F-1, 5F-2, and 5F-3) to amplify the desired 5′ flanked region. Similarly, only arbitrary primer BOO43 was successfully used to pair with the other set of nested specific primers (3Z-1, 3Z-2, and 3Z-3) to amplify the 3′ flanked region. After sequence assembly, a full B3-LAAO-coding sequence with 1608 bps in length was obtained. This open reading frame (ORF) encodes 535 amino acid (AA) residues with a predicted molecular mass of 59571.46 Da (60 kDa). A BLASTN search of the ORF nucleotide sequence at the NCBI website showed that it shared 75% and 73% identity to L-aspartate oxidase from *Pseudoalteromonas *sp. SM9913 and *Shewanella denitrificans* OS217, respectively. According to the deduced amino acid sequence, further BLASTP search at the NCBI website revealed that B3-LAAO had the highest homology with a number of L-aspartate oxidases, giving 80%, 80%, 80%, 73%, and 70% identity to L-aspartate oxidases from *Pseudoalteromonas *sp. SM9913, *Pseudoalteromonas haloplanktis *ANT/505, *Pseudoalteromonas tunicata *D2, *Alishewanella jeotgali *KCTC 22429, and *Shewanella denitrificans* OS217, respectively. So far, most of LAAOs found in *Pseudoalteromonas* genus have a strict preference for specific substrate, for example, L-aspartate oxidase from *Pseudoalteromonas *sp. SM9913, *Pseudoalteromonas haloplanktis *ANT/505 and *Pseudoalteromonas tunicata* D2, and L-lysine oxidase from *Pseudoalteromonas tunicate* D2 [17]. In contrast, B3-LAAO exhibited a broad substrate spectra with the highest activity to L-Leu, followed by L-Lys, L-Tys, L-Asn, L-Gln, L-Met, L-cystine, L-Arg, L-Trp, and L-Glu but no detectable activity to L-aspartic acid (data not shown). So that B3-LAAO could represent a novel LAAO, which can certainly broaden our view on LAAO from *Pseudoalteromonas* genus. Based on the alignment of amino acid sequences of 6 homologous LAAOs (see Figure 4), B3-LAAO gave two highly conserved motifs. One is the dinucleotide-binding motif ohhhhGxGxxGxxxhxxL which is a typical FAD binding site where o stands for a polar or charged residue and h for a hydrophobic residue; the other is the characteristic sequence motif RxGG. These motifs appear in several families of the flavoproteins [1, 8].

### 3.4. Expression of B3-LAAO in *E. coli* BL2 (DE3)

**Figure 5:**
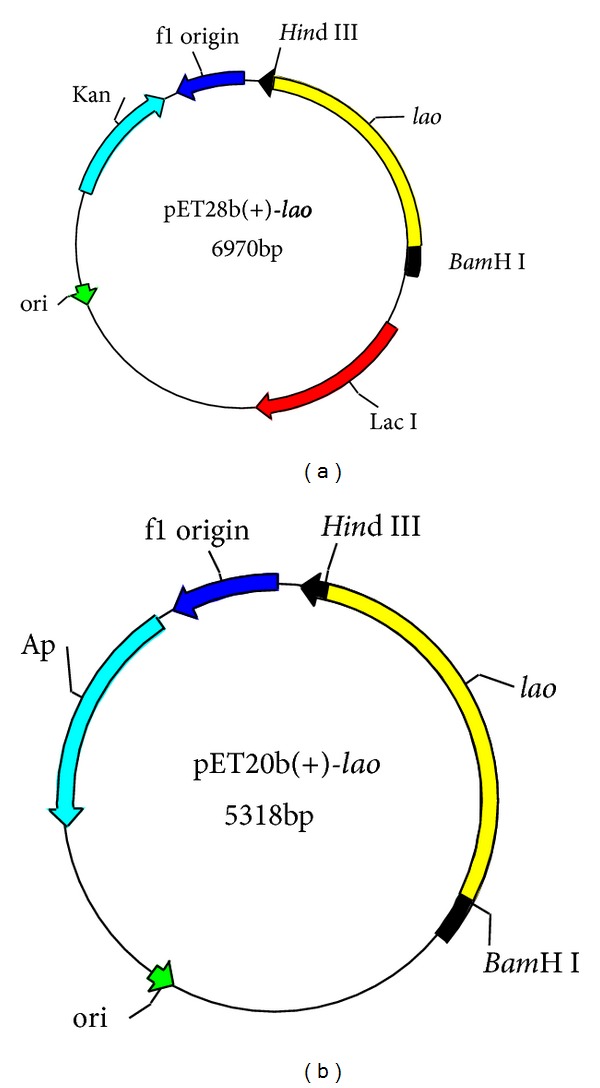
Maps of the recombinant plasmids containing *lao*-gene, pET28b(+)-*lao* (a), and pET20b(+)-*lao* (b). The LAAO-encoding gene was cloned between *Bam*H I and *Hin*d III restriction sites.

**Figure 6:**
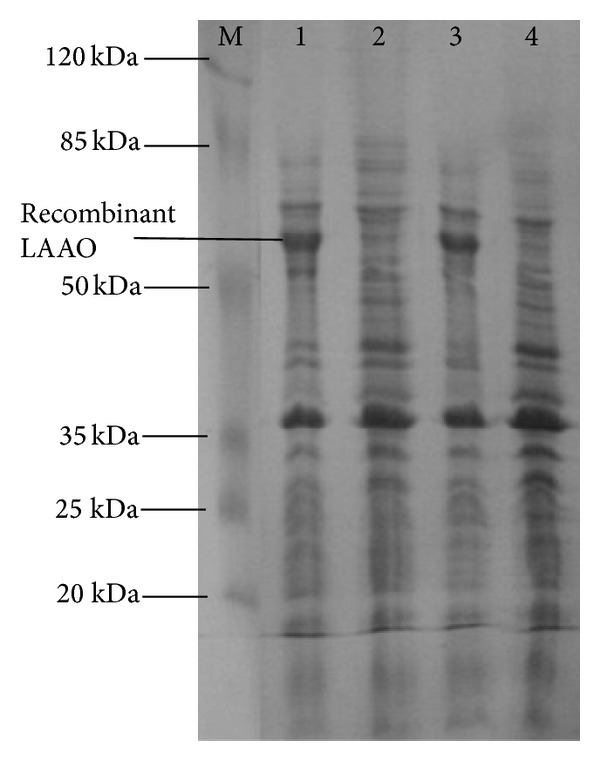
SDS-PAGE analysis of the expressed recombinant B3-LAAO in *E. coli* BL21(DE3). Lane M: prestained protein marker (Thermo, USA); lane 1: cell culture of *E. coli* BL21(DE3)/pET28b(+)-*lao* with a 6 h-induction by IPTG; lane 2: cell culture of *E. coli* BL21(DE3)/pET28b(+)-*lao* without induction; lane 3: cell culture of *E. coli* BL21(DE3)/pET20b(+)-*lao* with a 6 h-induction by IPTG; Lane 4: cell culture of *E. coli* BL21(DE3)/pET20b(+)-*lao *without induction.

Attempts to express B3-LAAO in* E. coli *were made and two different expression vectors pET-28b(+)-*lao* and pET-20b(+)-*lao* were constructed for this purpose (see Figure 5). The *lao*-gene was cloned into pET28b(+) and pET20b(+), respectively, and both were separately transformed into* E. coli* BL21(DE3) strain and expressed as described in Section 2. PCR amplification, restriction endonuclease digestion, and direct sequencing analysis demonstrated that plasmids of pET28b(+)-*lao* and pET20b(+)-*lao* were successfully constructed (data not shown). SDS-PAGE analysis (see Figure 6) showed that the recombinant B3-LAAOs on the two different vectors both can be induced and expressed to give the same molecular mass of about 60 kDa, agreeing with the calculated one based on the deduced amino acid sequence.

**Figure 7:**
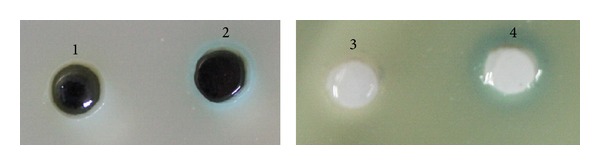
Measurement of the expressed B3-LAAO activity using Prussian blue agar assay [16]. Lane 1: *E. coli* BL21(DE3)/pET28b(+)-*lao *without induction; lane 2: *E. coli* BL21(DE3)/pET28b(+)-*lao *with a 6 h-induction; lane 3: *E. coli* BL21(DE3)/pET20b(+)-*lao *without induction; lane 4: *E. coli* BL21(DE3)/pET20b(+)-*lao *with a 6 h-induction.

The activity of the expressed recombinant LAAO was determined with Prussian blue agar assay [16] using L-Leu as substrate. The results in Figure 7 indicated that, after induction by IPTG, the recombinant B3-LAAOs in culture supernatants from *E. coli* BL21(DE3) transformed with both pET28b(+)-*lao *and pET20b(+)-*lao* can cause the Prussian blue agar to form blue halos which resulted from the H_2_O_2_ production, while no LAAO activity could be detected in all tested samples from the sonicated cell pellets (data not shown). In fact, accumulation of inclusion bodies was found in cell pellets. These inclusion bodies could be dissolved in 8 M urea, but the enzyme activity could not be restored by slowly diluting the solution. All these findings indicate that B3-LAAO could functionally be expressed in *E. coli*, but the expressed level was relatively low. The following reasons may account for this phenomenon. First, H_2_O_2_ produced by LAAO activity is toxic to *E. coli* [18], and thus this would likely inhibit the LAAO expression level; second, the recombinant LAAO from *E. coli* may be much less active than the wild-type one; third, *E. coli* may be a relatively unsuitable host for the heterologous production of B3-LAAO. Several ways may give solutions to these common problems. First, other hosts can be used to replace *E. coli* for heterologous expression of LAAO [9]. Second, coexpression of chaperone protein may prevent the aggregation of the recombinant LAAO in *E. coli* [19]. Third, the expression conditions can be varied, considering the inductor concentration, time of induction, induction temperature, and duration, to reduce the amount of insoluble fractions. Last but not least, inclusion bodies can be dissolved into urea for restoration of its activity through dialysis.

It has been found that the change in diameter of blue halo on Prussian blue agar is a function of H_2_O_2_ concentration with exponential fit [16]. According to the size of halos, Figure 7 further showed that *E. coli* BL21(DE3)/pET20b(+)-*lao* with a signal peptide had the higher activity than *E. coli *BL21(DE3)/pET28b(+)-*lao* without signal peptide. Most probably, secretion of recombinant LAAO can help to reduce the harm of intracellular active LAAO to cells.

## 4. Conclusion

In the present study, an LAAO-producing marine bacterium B3 was successfully isolated from the intertidal sea area. 16S rDNA sequence and phylogenetic tree analysis together with physiological and biochemical assays revealed that it can be designated as *Pseudoalteromonas* sp. B3. Its capability of LAAO production was demonstrated by determining the release of H_2_O_2_, *α*-keto leucine, and NH_4_^+^ after oxidization of L-Leu. Through two rounds of PCR, entire LAAO-coding gene sequence from *Pseudoalteromonas* sp. B3 was obtained. Its deduced amino acid sequence of 535 residues showed two conserved motifs, GG-motif and *βαβ*-dinucleotide-binding domain motif, typical to a number of flavoproteins. B3-LAAO had the highest identity of only around 80% to several L-aspartate oxidases from different microorganisms. In addition, it had high activities to broad L-amino acids but not to L-aspartic acid. Therefore, B3-LAAO probably presents a novel LAAO, which can enrich our knowledge on LAAO from *Pseudoalteromonas *genus.

To date, heterologous expression of LAAO is still a big challenge due to its toxicity to host and the requirement of posttranslational modification. In this study, B3-LAAO was successfully and functionally expressed in *E. coli* BL21(DE3) using two different vectors pET28b(+) and pET20b(+). It was found that addition of suitable concentration FAD and Zn^2+^ during IPTG induction can increase the activity of recombinant B3-LAAO. Besides, lower induction temperature (25°C) was better for B3-LAAO expression than higher induction temperature (37°C). Most probably, low temperature can reduce the aggregation of B3-LAAO. In future, further lower induction temperature (16°C) is highly promising. No active intracellular LAAO activity was detected, but huge inclusion bodies were formed in cell, which is common in heterologous expression of LAAO [9, 11]. Overall, the expression level of active B3-LAAO was relatively low. We believe that this level is only for soluble LAAO, while much of the LAAO is present in insoluble inclusion bodies. Therefore, in future, the expression conditions will be optimized, considering the inductor concentration, time of induction, induction temperature, and duration. In addition, the inclusion bodies will be dissolved into urea for its activity restoration. Finally, coexpression of chaperone protein with B3-LAAO in *E. coli* is also being performed in our lab.

## Figures and Tables

**Table 1:** Conserved regions of LAAOs from common marine microorganisms used for design of degenerate primers.

Item	Accession no.	Sequences of^a^	
		Region 1	Region 2
*Comamonas testosteroni *KF-1	ZP_03541004	58**VLGAGL**AGM66	333**ADWCVCT**IP341
*Dinoroseobacter shibae* DFL 12	ABV95616	59**VLGAGL**AGM67	337**ADWCVCT**IP345
*Caulobacter* sp. K31	YP_001683007	57**VLGAGL**AGM65	331**ADWCVCT**IP345
Degenerate primer		ZZ-1: 5′-GTGCTSGGCGCKGGYCT-3′	ZF-1: 5′-GTGCARACGCASYAGTCG-3′

^a^Boldface letters indicate the specific amino acids actually used in the design of the degenerate primers (forward primer: ZZ-1; reverse primer: ZF-1).

**Table 2:** Conserved regions of LAAOs from *Pseudoalteromonas* microorganisms used for design of degenerate primers.

Item	Accession no.	Sequences of^a^		
		Region 1	Region 2	Region 3
*Pseudoalteromonas haloplanktis* TAC125	YP_339251	13**IIGSGAA**AGLS22	225**AMAWRAG**C232	359**MTDFNGK**TDL368
*Pseudoalteromonas tunicata *D2	ZP_01132853	13**IIGSGAA**AGLS22	224**AMAWRAG**C231	357**MTDFNGK**TDL366
*Pseudoalteromonas atlantica *T6c	YP_662716	13**IIGSGAA**AGLS22	222**AMAWRAG**C230	359**VTDFNAK**TDL368
*Pseudoalteromonas *sp. SM9913	YP_004069407	13**IIGSGAA**AGLS22	225**AMAWRAG**C232	359**MTDFNGK**TDL368
Degenerate primer^b^		ZZ-2: 5′-ATTATHGGYAGCGGCGC-3′	ZZ-3: 5′-GCHATGGCDTGGCGTGC-3′ ZF-2: 5′-CCHGCACGCCAHGCCAT-3′	ZF-3: 5′-TDSCATTAAAGTCRGTC-3′

^a^Boldface letters indicate the specific amino acids actually used in the design of the degenerate primers (forward primers: ZZ-2 and ZZ-3; reverse primers: ZF-2 and ZF-3).

**Table 3:** Primers used in cloning of 5′ and 3′ flanked sequences of LAAO gene from *Pseudoalteromonas *sp. B3.

Primers*	Sequences (5′-3′)	Size (bp)
Set A		
5F-1	TTGCCAAAGACAGAGCCAGGCT	22
5F-2	AGGTCGCCCTTACTGAGCACTGT	23
5F-3	TCTTATCAAACACCGCAGCAAT	22
Set B		
3Z-1	ATTGCCATGGCTTGGCGTG	18
3Z-2	GTCAAATCCAGACGTTTCCAGT	22
3Z-3	GCGGTGCCAGTAAAGTGTATCT	22
Set C		
S-1	ACGATGGACTCCAGAGCGGCCCGCVNVNNNGGAA	34
S-2	ACGATGGACTCCAGAGCGGCCCGCBNBNNNGGTT	34
S-3	ACGATGGACTCCAGAGCGGCCCGCHNVNNNCCAC	34
S-4	ACGATGGACTCCAGAGCGGCCCGCVVNVNNNCCAA	35
S-5	ACGATGGACTCCAGAGCGGCCCGCBDNBNNNCGGT	35
B0043	NNNNNN	6
B0043-9	NNNNNNNNN	9
B0043-10	NNNNNNNNNN	10

*Primers in set A are one set of nested specific primers for amplification of 5′ flanked region of B3-LAAO gene; primers in set B are one set of nested primers for amplification of 3′ flanked region of B3-LAAO gene; primers in set C are arbitrary primers for amplification of 5′ and 3′ flanked regions of B3-LAAO gene.

**Table 4:** Detection of the H_2_O_2_ production using Amplex Red Hydrogen Peroxide/Peroxidase Assay kit.

Reactions	Amount of H_2_O_2 _(mM)	Relative activity (%)
Test 1^a^	1.84	100
Test 2^b^	0.14	7.61
Test 3^c^	0.043	2.34

^a^Culture supernatant of strain B3 with substrate L-Leu; ^b^culture supernatant of strain B3 without substrate L-Leu; ^c^same as Test 1, except that the culture supernatant of strain B3 was denatured with boiling before addition of substrate L-Leu.
